# Proximity Proteomics Reveals USP44 Forms a Complex with BRCA2 in Neuroblastoma Cells and Is Required to Prevent Chromosome Breakage

**DOI:** 10.3390/biomedicines12122901

**Published:** 2024-12-20

**Authors:** Asma Ali, Sajjad Hussain, Tibor Bedekovics, Raymond H. Jeon, Danielle G. May, Kyle J. Roux, Paul J. Galardy

**Affiliations:** 1 Department of Pediatric and Adolescent Medicine, Mayo Clinic, Rochester, MN 55905, USA; ali.asma@mayo.edu (A.A.); hussain.sajjad@mayo.edu (S.H.); bedekovics.tibor@mayo.edu (T.B.); raymondhjeon@gmail.com (R.H.J.); 2 Department of Family Medicine, Mayo Clinic, Rochester, MN 55905, USA; 3 Enabling Technology Group, Sanford Research, Sioux Falls, SD 57104, USA; danielle.may@sanfordhealth.org (D.G.M.); kyle.roux@sanfordhealth.org (K.J.R.); 4 Department of Pediatrics, Sanford School of Medicine, University of South Dakota, Sioux Falls, SD 57105, USA; 5 Division of Pediatric Hematology-Oncology, Mayo Clinic, Rochester, MN 55905, USA

**Keywords:** neuroblastoma, proteomics, ubiquitin, Fanconi anemia

## Abstract

Background/Objectives: The enzyme ubiquitin-specific protease 44 (USP44) is a deubiquitinating enzyme with identified physiological roles as a tumor suppressor and an oncogene. While some binding partners and substrates are known for USP44, the identification of other interactions may improve our understanding of its role in cancer. We therefore performed a proximity biotinylation study that identified products of several known cancer genes that are associated with USP44, including a novel interaction between BRCA2 and USP44. Methods: We expressed a fusion protein that linked USP44 and mutant Escherichia coli biotin ligase BioID in SH-SY5Y neuroblastoma cells. Control experiments were performed using BioID alone. In duplicate experiments, cells were pulsed with biotin and biotinylated proteins were isolated under denaturing conditions and the proteins were identified by mass spectrometry. The resulting list of proteins were analyzed using Enrichr and cross-referenced with the COSMIC Cancer Gene Census. We validated the association with BRCA2 using immunoprecipitation. The role of USP44 in the Fanconi anemia DNA repair pathway was investigated using chromosome analysis of wild-type or Usp44-knockout cells after exposure to mitomycin C. Results: We identified 146 proteins that were selectively retrieved by the USP44 construct and compared with cells expressing the BioID ligase alone, including 15 gene products encoded by genes on tier 1 of the COSMIC Cancer Gene Census, including BRCA2. The association between USP44 and BRCA2 was validated through immunoprecipitation. We tested the functional role of USP44 in the Fanconi anemia DNA repair pathway through chromosome breakage analysis and found that cells lacking USP44 had a significant increase in chromosome breaks and radial chromosomes. We found that high *BRCA2* transcript was correlated with poor survival in neuroblastoma, likely due to its tight association with proliferation in these tumors. Conclusions: Our results identified novel potential binding partners and potential substrates for USP44, including several with direct roles in cancer pathogenesis. Our results identified a novel association between BRCA2 and USP44, and a previously unknown role for USP44 in the Fanconi anemia DNA repair pathway that may contribute to its role in cancer.

## 1. Introduction

The ubiquitin proteasome pathway has long been known to be an important cellular pathway that regulates many signaling events essential to the promotion of, or protection from, cancer. We initially became interested in USP44 following its identification as a mediator of the spindle assembly checkpoint that protects cells from errors in mitosis and the development of aneuploidy [1]. Subsequently, we and others have found USP44 to contribute to the pathophysiology of several cancers, with roles as an oncogene and tumor suppressor depending on the cellular context of its deregulation [2,3,4,5,6,7,8,9,10,11,12]. Our mechanistic studies suggest that the participation of USP44 in these various pathways may depend on its specific domains that differentially mediate interaction with binding partners and substrates. We found that the role of USP44 as a tumor suppressor is, in part, dependent on its binding to the centriole protein centrin—a complex that is required for proper timing of centrosome separation and DNA repair through the nucleotide excision repair pathway [11,13]. Recently, we also identified a pro-tumorigenic role that involves USP44 deubiquitinating histone H2B [2,14]. This function is hypothesized to rely on association with the N-CoR nuclear co-repressor [14]. While multiple potential mechanisms have been identified to mediate the roles of USP44 in cancer, to our knowledge there has been no unbiased examination of the USP44 interactome to help uncover oncogenic or tumor-suppressive mechanisms.

Here, we describe work in which we utilized proximity proteomics to develop an interaction map of USP44. We expressed a fusion protein that linked USP44 with the promiscuous E. coli BirA^R118G^ biotin ligase (BioID) that generates a cloud of biotinylation approximately 10 nm in diameter [15]. In the context of our recent work, in which we found USP44 to promote the malignant phenotype of the childhood cancer neuroblastoma, we performed this proteomic analysis in the widely used neuroblastoma cell line SH-SY5Y. When compared with those proteins isolated from cells expressing BioID alone, we identified 146 proteins in the proximity of USP44, including many known cancer-related proteins. We validated the association of USP44 with BRCA2, one of the proteins identified in our study—uncovering a previously unknown function of USP44 in the Fanconi anemia DNA repair pathway.

## 2. Materials and Methods

### 2.1. Proximity Proteomics

The BioID biotin ligase [16] was cloned as an N-terminal fusion with the human USP44 cDNA into the TSIN lentiviral vector [17] using standard techniques. SH-SY5Y neuroblastoma cells, obtained from the ATCC, were stably transduced with lentivirus encoding the BioID-USP44 construct, or BioID alone. Biotinylation and BioID pulldowns were performed in duplicate as described [18].

**Sample preparation for MS.** After immunoprecipitation, a digestion was performed with proteins on the beads. Briefly, the beads were resuspended in a solution containing 8 M urea, 50 mM ammonium bicarbonate, and 10 mM tris(2-carboxyethyl)phosphine (TCEP). The mixture was incubated at 30 °C for 60 min followed by an additional incubation at 21 °C for 30 min in the dark after the addition of 30 mM iodoacetamide (IAA). Then, 50 mM ammonium bicarbonate was added to dilute to 1 M urea and the proteins were digested overnight with Mass Spec Grade Trypsin/Lys-C Mix (Promega, Madison, WI, USA). The mixture was then centrifuged, and the supernatant was transferred to a new tube. The beads were washed once with 50 mM ammonium and the supernatant added to the digested proteins. The solution was desalted using AssayMap C18 cartridges mounted on a BRAVO liquid handling system (Agilent, Columbia, MD, USA), and the organic solvent was removed in a SpeedVac concentrator prior to LC-MS/MS analysis.

**LC-MS/MS analysis.** After reconstituting the samples with 2% acetonitrile and 0.1% formic acid, analysis was performed by LC-MS/MS on a Proxeon EASY nanoLC system (Thermo Fisher Scientific, Waltham, MA, USA) coupled to an Orbitrap Elite mass spectrometer (Thermo Fisher Scientific) operated in positive data-dependent acquisition mode. An analytical column (C18 Acclaim PepMap; 0.075 × 500 nm, 2 µm particles; Thermo Scientific) was used for peptide separation with a 180 min gradient of 2–28% solvent B at a flow rate of 300 nL/min. MS1 spectra were measured at a resolution of 60,000, an 1 × 10^6^ AGC target, and a mass range from 350 to 1400 *m*/*z*. Up to 10 MS2 spectra per duty cycle were triggered, fragmented by collision-induced dissociation, and acquired in the ion trap with an AGC target of 1 × 10^4^, an isolation window of 2.0 *m*/*z,* and a normalized collision energy of 35. Dynamic exclusion was enabled with a duration of 30 s. 

**Data analysis.** Spectra were analyzed using MaxQuant software version 1.5.5.1 against the Homo sapiens Uniprot protein sequence database (version July 2016) and GPM cRAP sequences (commonly known protein contaminants). Precursor mass tolerance was 20 ppm for the first search where initial mass recalibration was completed, and then 4.5 ppm for the main search. Mass tolerance was set to 0.5 Da for the product ion search. The maximum precursor ion charge state used for searching was 7. Carbamidomethylation of cysteines was searched as a fixed modification, while oxidation of methionines and acetylation of the protein N-terminal were searched as variable modifications. A maximum of two missed cleavages was set in the search using trypsin as the enzyme in a specific mode. The target-decoy-based false discovery rate (FDR) filter for spectrum and protein identification was set to 1%.

### 2.2. Bioinformatic Analyses and Statistics 

The list of retrieved proteins from proximity proteomics was analyzed using the enrichr web tool (Enrichr (maayanlab.cloud)), as described [2,19,20,21]. An interaction network was generated using the STRING online tool (https://string-db.org/) accessed 1 November 2024. The analysis was performed with settings to include the ‘physical subnetwork’; network edges set to ‘evidence’; active interaction sources including only ‘experiments’; minimum required interaction score set to ‘medium evidence (0.400)’; maximum number of interactors set to ‘none/query proteins only’ for the 1st shell and ‘none’ for the second shell; and advanced settings set to ‘interactive’, ‘enable 3D bubble design’, ‘center protein names on nodes’, and ‘hide disconnected nodes in the network’. Clinically annotated gene expression datasets were analyzed initially through the R2: Genomics analysis and visualization platform (http://r2.amc.nl) (accessed 1 November 2024). Data are from the SEQC dataset (GSE62564), Primary NRC (GSE85047) and TARGET (https://ocg.cancer.gov/programs/target) (accessed 1 November 2024). The Whitfield proliferation signature score was calculated by generating a custom gene set utilizing the list of 45 genes from [22] within R2 and utilizing the ‘sample ranked genesic scores’ function from the ‘small tools’ menu within R2. For preparation of graphs, data were extracted from R2 using the datagrabber function into a Microsoft Excel spreadsheet. Data were then sorted and transferred to GraphPad Prism software 10.3.1. Statistical calculations were performed using the embedded analysis tools in Prism.

### 2.3. Cell Culture, Immunoprecipitation, and Immunoreagents

SH-SY5Y cells were maintained in DMEM with 20% fetal calf serum. Cells were transduced with lentivirus-encoding hemagglutinin (HA)-tagged human USP44 with or without GFP-tagged BRCA2 (a kind gift from Dr. Zhenkun Lou). *Usp44-*wild-type and *Usp44-*null murine embryonic fibroblasts (MEFs) immortalized with the SV40 large T antigen were generated and cultured as described previously [11,12]. Total-cell lysates were prepared for immunoprecipitation in 1 mL of NP-40 lysis buffer (1% Nonidet P-40 (NP-40), 50 mM Tris-HCl (pH 8.0), 150 mM NaCl, 5 mM EGTA, 5 mM EDTA, 15 mM MgCl2, 60 mM b-glycerolphosphate, 1 mM dithiothreitol, and 1 tablet of protease inhibitor cocktail per 10 mL (Roche, cOmplete Mini, Basel, Switzerland). Lysates were kept on ice for 10 min, vortexed gently at 2 min intervals, and centrifuged for 5 min at 14,000× *g*. The supernatants were filtered through a 0.45 mm-pore-size low-protein-binding syringe filter (Acrodisc; Millipore, Burlington, MA, USA) and precleared with 100 μL of protein A/G-sepharose (50% slurry in NP-40 lysis buffer; Thermo Fisher) for 1.5 h at 4 °C with rotation. Primary immunoprecipitations were performed by adding 50 μL of (50% slurry) of anti-HA high-affinity matrix (Roche). Following a 3 h rotation at 4 °C, the pellets were washed five times in NP40 lysis buffer and antigens were released by boiling for 2 min in 100 μL of SDS sample buffer (BioRad, Hercules, CA, USA). Precipitates were separated by SDS-PAGE and immunoblots were probed as indicated. Immunoreagents included anti-HA high affinity antibody (3F10-HRP conjugated; Roche), anti-HA high affinity matrix (Roche), anti-GFP (Abcam; ab290, Cambridge, UK), and anti-BRCA2 (A303-435; Millipore). Secondary antibodies conjugated to HRP were purchased from Cell Signaling Technology.

### 2.4. Chromosome Analysis

Wild-type (*Usp44*^+/+^) or *Usp44-*null (*Usp44*^−/−^) cells were transduced or not with lentivirus-encoding USP44-HA. Cells were incubated for 48 h with varying concentrations of mitomycin C, and metaphase spreads were prepared and analyzed as described [23]. Briefly, cells recovered from a 25 cm^2^ flask were incubated with colcemid (0.05 μg/mL) for 6 h. After collecting the media to capture any floating cells, adherent cells were collected using trypsin EDTA. The cells were then centrifuged and resuspended in 5 mL pre-warmed (37 °C) hypotonic solution (0.075 M KCl in deionized water) and cells were incubated at 37 °C for 10 min. Five drops of Carnoy’s fixative (3:1 ratio methanol:acetic acid) were added followed by gentle inversion. Cells were centrifuged and resuspended in 5 mL Carnoy’s fixative and then incubated at room temperature for 10 min, after which cells were washed three times with resuspension in fixative each time. After the final wash, cells were resuspended in 300 μL fixative and dropped onto slides from a height of approximately 30 cm. Slides were stained with Giemsa solution and visualized by light microscopy.

## 3. Results

**Figure 1:**
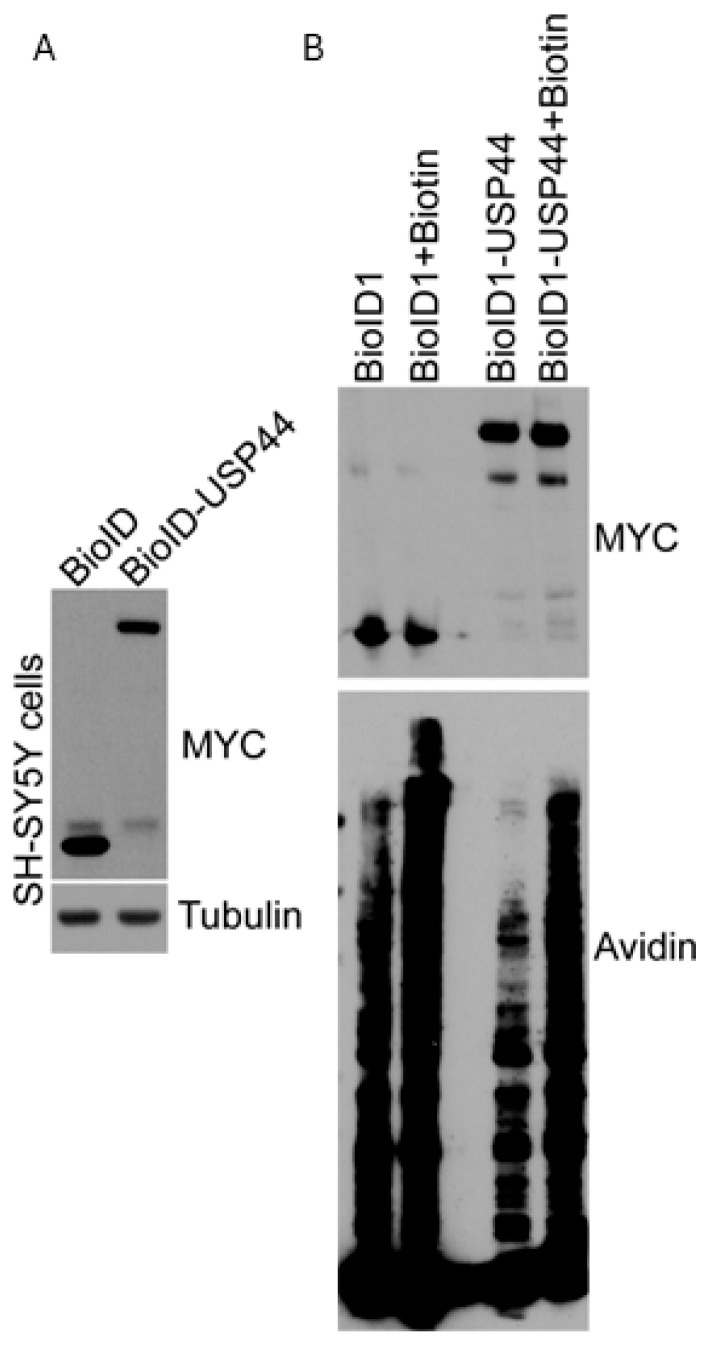
Expression of BioID or BioID-USP44 in SH-SY5Y cells. (**A**) Cells of the neuroblastoma cell line SH-SY5Y were transduced with lentivirus encoding the BioID biotin ligase alone, or as an N-terminal fusion with USP44. The expression of both was monitored by immunoblotting to detect the MYC-tag encoded in BioID. (**B**) SH-SY5Y cells as in (**A**) were incubated with biotin for 16 h and the presence of biotinylated proteins was monitored by probing blots with streptavidin-HRP.

To identify novel binding partners or substrates for USP44, we utilized the BioID proximity proteomics approach [24]. We aimed to discover USP44-interacting proteins without regard to pre-existing knowledge of their role within the cell in the hope of uncovering novel mechanisms in normal or pathologic cell biology. The proximity proteomics approach was selected due to several advantages. Firstly, the expression of the bait within cells leads to its physiological localization. The biotinylation process then occurs in this location—avoiding the identification of interactions that only occur after cell lysis, when proteins that may not normally co-localize may now be exposed to each other. Secondly, the strong interaction between biotin and streptavidin—and the stability of streptavidin—allows for the isolation of interacting proteins in very harsh denaturing conditions, to reduce non-specific interactions. As we recently found USP44 over-expression to promote aggressive features in neuroblastoma, we used the SH-SY5Y neuroblastoma cell line for this study. We generated a fusion construct in which a cDNA encoding the BioID biotin ligase was cloned in-frame with a cDNA encoding human USP44. The resulting construct, as well as a control expressing the BioiD ligase alone, was cloned into the pTSIN lentivirus expression vector, and SH-SY5Y neuroblastoma were transduced to produce polyclonal stable pools. The expression of the constructs was verified by immunoblotting (Figure 1A). The cells were then pulsed with biotin for 16 h to induce biotinylation of proteins in the proximity of the expressed bait (Figure 1B). Taking advantage of the strong interaction between biotin and streptavidin, the resulting biotinylated proteins were isolated using streptavidin beads under denaturing conditions in order to reduce the identification of non-specific interactions. The recovered proteins were then analyzed by mass spectrometry. The abundance of the identified proteins was quantified and compared between cells expressing each construct.

**Figure 2:**
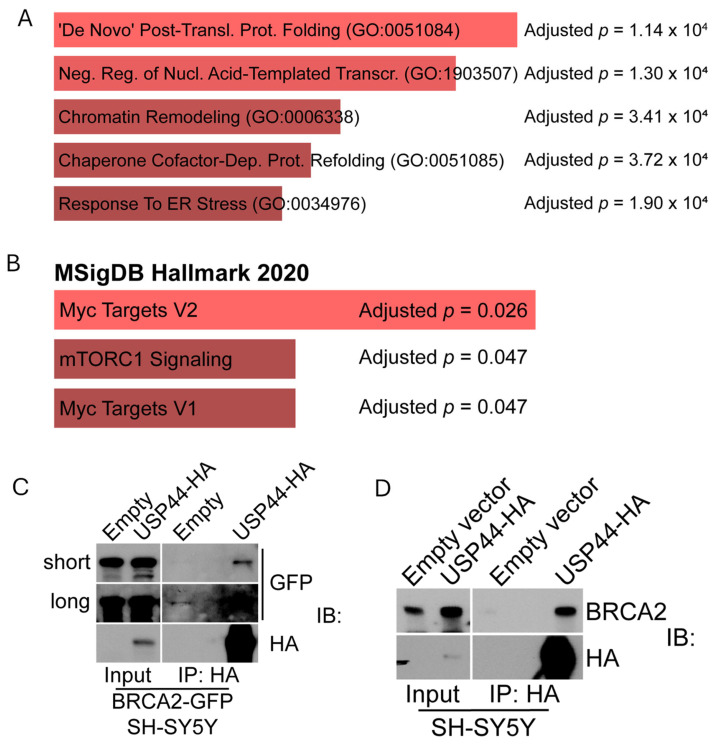
The proximity proteome of USP44 identified a novel function in the Fanconi anemia DNA repair pathway. (**A**,**B**) Enrichr analysis using the list of biotinylated proteins recovered (enrichment > 3-fold) from SH-SY5Y cells expressing the USP44-BioID fusion compared with those expressing BioID alone. Adjusted *p*-values (correction for multiple hypothesis testing) are shown. (**C**,**D**) SH-SY5Y cells expressing BRCA2-GFP (**C**) or not (**D**) were transduced with lentivirus-encoding USP44-HA followed by HA immunoprecipitation and immunoblotting for the indicated proteins. In (**C**), a short or long exposure of the blot was performed as indicated. The results are representative of at least three independent experiments.

A total of 146 proteins were enriched at least 3-fold comparing cells expressing the USP44-BioID fusion compared with those expressing the BioID fusion alone, 90 of which were recovered only from USP44-BioID-expressing—and not from BioID-expressing—cells (Table S1). Known USP44-interacting proteins, including centrin 2/3 and NCOR1/2, were retrieved uniquely with the USP44 fusion construct—validating this approach [11,14]. We analyzed the list of retrieved proteins using online tool Enrichr (Enrichr; https://maayanlab.cloud/Enrichr/) (accessed 1 November 2024) [21,22,23], focusing on gene ontology (GO) biological processes, and Hallmark gene sets (Figure 2A,B). Of the top five enriched GO processes, three involved protein folding, chaperones, and response to ER stress. It is unknown if USP44 had a role in these events or whether their retrieval was a byproduct of the fusion protein interacting with these proteins during synthesis. The remaining two pathways were related to transcription and chromatin remodeling—consistent with known functions of USP44 as part of the N-CoR complex [14]. The list of proteins retrieved by the BioID-USP44 fusion protein included three gene sets from the ‘Hallmark’ collection from the MsigDB collection, including two MYC targets gene sets (Figure 2B). Recently, we found that USP44 promotes a MYC-like gene expression signature in mouse embryonic fibroblasts and human neuroblastoma cells.

To further evaluate its role of in cancer, we cross-referenced the USP44 proximity proteome data with the Cancer Gene Census curated by the Catalogue of Somatic Mutations in Cancer (COSMIC|Catalogue of Somatic Mutations in Cancer (sanger.ac.uk) [25]). Of the 146 proteins retrieved from SH-SY5Y neuroblastoma cells, there were 13 tier-one cancer genes from this database (Table S2). [2]. To better understand how the USP44 proximity proteome may work as a network, we utilized the STRING protein interaction database [26]. The largest network is comprised of several gene products related to protein folding and chaperones—consistent with the enrichment data in Figure 2 (Figure S1). As expected, based on known interaction data, USP44 formed a cluster with NCOR1 and NCOR2, as well as CETN2 and CETN3. Two of the cancer gene products in the proximity proteome are known to physically interact with the N-CoR complex (SMARCE1 and SPEN)—suggesting these may all operate through a similar mechanism. Another large complex identified was centered on POL2RA and included an indirect interaction with KMT2A proteins important in transcription and its regulation via transcription factors and epigenetic regulation. Finally, a four-protein network centered on the protein CD2BP2 was observed. This protein is a known splicing factor associating with—and facilitating the maturation of—the U5-52K spliceosome [27,28,29]. This factor has recently been shown to be essential in a mouse knockout model for T-cell homeostasis [30].

**Figure 3:**
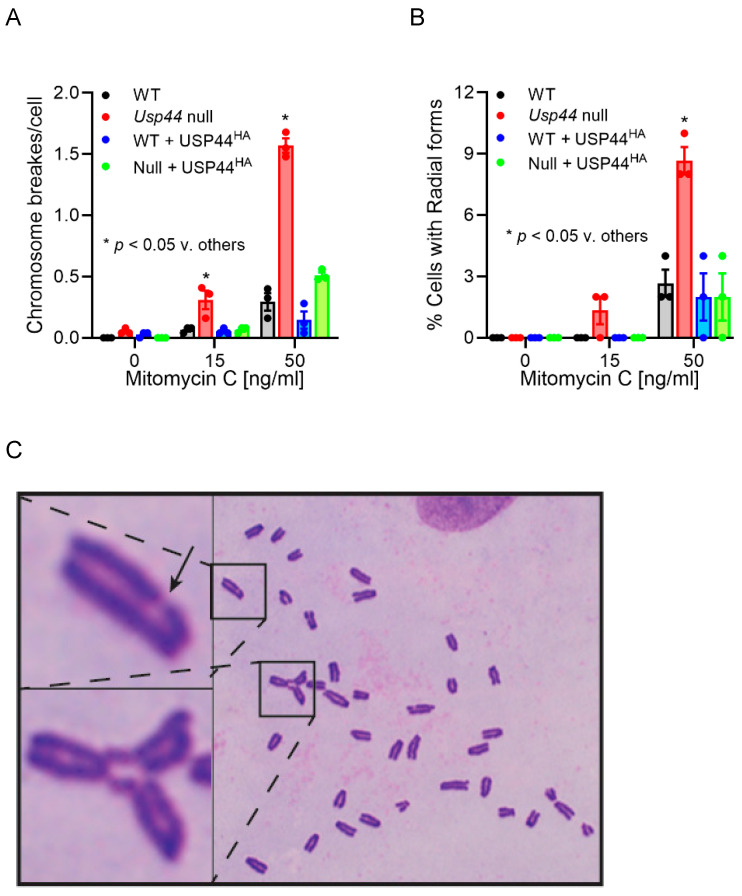
The proximity proteome of USP44 identifies a novel function in the Fanconi anemia DNA repair pathway. (**A**,**B**) Chromosome analysis was performed on colcemid-prepared metaphase preparations from murine embryonic fibroblasts (MEFs) with the indicated genotypes, with pre-treatment with the indicated concentrations of mitomycin C. The graphs represent the means +/− SEM of three independent MEF lines (prepared from independent embryos) and depict the number of chromosome breaks (**A**) or radial chromosome forms (**B**) *p*-values calculated using an unpaired Student’s *t*-test comparing *Usp44-*null to the other conditions at each concentration of MMC. (**C**) A representative metaphase spread from a *Usp44-*null culture treated with mitomycin C with a chromosome break (upper inset; indicated by arrow) and radial chromosome form (lower inset).

Due to its importance in DNA damage repair and transcription, and its general importance as a tumor suppressor gene, we sought to validate the interaction between USP44 and BRCA2. We found that HA-tagged USP44 co-precipitated both GFP-tagged and endogenous BRCA2 from SH-SY5Y neuroblastoma cells (Figure 2C,D). We did not observe a change in the level of BRCA2 protein in the presence or absence of USP44 over-expression. To examine the functional implication of the interaction between USP44 and BRCA2, we made use of *Usp44-*null mouse embryonic fibroblasts (MEFs) [11]. Loss of *BRCA2* (also known as *FANCD1*) leads to a cellular phenotype that includes increased chromosome breakage and the formation of radial chromosome forms following exposure to mitomycin C (MMC) [31,32]. We therefore performed karyotype analysis of *Usp44* wild-type and -null MEFs after exposure to varying concentrations of MMC. We found a significant increase in chromosome breaks and radial forms seen in cells lacking USP44 that was rescued with the re-introduction of USP44 (Figure 3A–C). We conclude from this data that USP44 forms a complex that includes BRCA2 and that USP44 is required to prevent MMC-induced chromosome aberrations. The data strongly suggest that USP44 works through BRCA2 in this process, but a direct mechanistic connection is not possible with our data.

**Figure 4:**
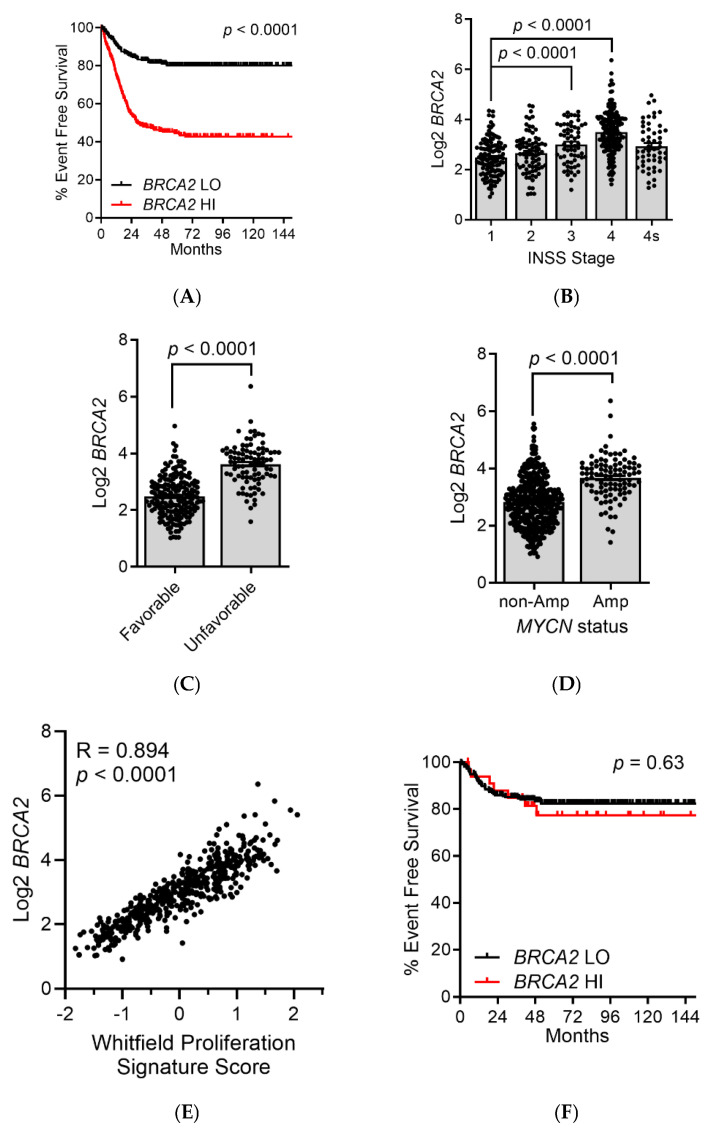
High *BRCA2* predicts poor outcomes and rapid proliferation in neuroblastoma. (**A**) The event-free survival is shown for patients in the SEQC dataset (*n* = 498) stratified by *BRCA2* expression (median). *p*-value calculated with the log-rank test. (**B**–**D**) Mean *BRCA2* transcript level is shown in patients from SEQC dataset separated by clinical stage (**B**), Shimada histology (**B**), and *MYCN* gene status (**C**). *p*-values calculated using the unpaired *t*-test. (**E**) Correlation between the transcript level of *BRCA2* and the Whitfield proliferation signature score. Each dot represents the BRCA2 level and the proliferation signature score for an individual tumor. The R-value represents the Pearson correlation value, and the corresponding 2-tailed *p*-value. (**F**) The event-free survival is shown for patients from the SEQC dataset with low Whitfield proliferation signature scores (<median; *n* = 249) stratified on BRCA2 expression (median).

We next wondered whether the interaction between USP44 and BRCA2 may have implications for survival in patients with neuroblastoma. Reasoning that USP44 promotes the activity of BRCA2, we hypothesized that high levels of *BRCA2* may independently associate with outcomes in neuroblastoma. We first examined event-free survival in the SEQC cohort (*n* = 498) for patients with high (>median) or low (<median) *BRCA2* expression. There was a highly significant decrease in EFS in patients whose tumors had high expression of *BRCA2* (Figure 4A). A similar result was also observed in two independent neuroblastoma datasets (Primary NRC *n* = 283, TARGET *n* = 249; Figure S2). Consistent with this, *BRCA2* expression was highly correlated with higher clinical stage, histology, and *MYCN* amplification in the SEQC dataset. (Figure 4B–D). As *BRCA2* expression varies through the cell cycle, we hypothesized that high BRCA2 may be identify tumors with a higher proliferation rate. *BRCA2* is tightly correlated with a proliferation gene signature in neuroblastoma across the SEQC, Primary NRC, and TARGET datasets (Pearson R = 0.894, 0.870, and 0.876, respectively; Figure 4E), perhaps explaining the association with these measures of aggressive disease. Consistent with this, when the survival analysis was restricted to patients with low proliferation signature scores (<median) the level of *BRCA2* no longer correlated with the scores (Figure 4F).

## 4. Discussion

The ubiquitin proteasome pathway plays an important role in the pathophysiology of cancer. We have a longstanding interest in the role of deubiquitinating enzymes in cancer and have shown that the enzyme USP44 has both tumor-suppressive and oncogenic roles. This prompted us to take an unbiased approach to identify novel binding partners. We used the BioID-based proximity proteomics approach as it enables the identification of associations that form in the native environment of the cell [15,16]. As cell lysis was performed under fully denaturing conditions, non-specific and post-lysis interactions were minimized. A limitation of this technique, however, relates to the radius of biotinylation which cannot distinguish direct binding proteins from those bound indirectly through larger protein complexes [16,24,33]. This limitation is shared with other affinity proteomic approaches—and requires in vitro analysis to determine if binding is direct or indirect. Our results also could not distinguish proteins that may have been USP44 substrates from binding partners.

Our data suggest that USP44 forms complexes with several known cancer genes implicated in several different cancer types. According to our analysis using the STRING database, complexes including SPEN, SMARCE1, and the N-CoR complex have been observed previously in several studies, as reported in the BioGRID database. Notably, eight of the identified cancer genes are implicated in hematologic malignancies according to COSMIC. The association with the protein CD2BP2 is intriguing. This protein is essential for the maturation of the U5-SNP spliceosome [27,28,29] and when deleted is essential to the survival of T-cells in a mouse knockout model [30]. We and others have observed that high USP44 is present in T-cell lymphoblastic leukemia (T-ALL; [3,12]), and that USP44 plays a role in Treg development [34]. We speculate that the association of USP44 with CD2BP2 may play a role in T-cell ALL. We chose to validate the data through experiments involving BRCA2. *BRCA2* is a tumor suppressor best known for mutations associated with hereditary breast and ovarian cancer, but mutations are also associated with predisposition to other cancers including childhood leukemia and neuroblastoma [31,35]. In addition to its mutation in human cancers, BRCA2 is known to participate in the Fanconi DNA repair pathway, and some biallelic variants in *BRCA2* are found in patients with clinical Fanconi anemia [32]. We found that cells lacking USP44 have a chromosome breakage phenotype similar to that seen in patients with Fanconi anemia. While it is tempting to connect this phenotype to a complex involving USP44 and BRCA2, we also identified FANCI specifically retrieved from cells expressing USP44-BioID (Table S1). FANCI, like FANCD2, is monoubiquitinated by the core Fanconi complex—an event that is essential for DNA repair through this pathway [36]. Removal of monoubiquitin from both FANCD2 and FANCI is required for repair, but removal by the deubiquitinating enzyme USP1 is also required for repair [23,36,37,38]. If USP44 is also involved in deubiquitination of FANCI a similar phenotype would therefore be expected. We also found a very strong correlation between *BRCA2* transcript level and a proliferation gene expression signature in neuroblastoma. It therefore seems likely that the association between high *BRCA2* and poor survival in neuroblastoma is most likely a result of *BRCA2* marking cells with a high proliferative rate. It is not unknown if independently increasing BRCA2 through preventing its degradation would have any impact on the proliferation of these cells. Our results, indicating a role for USP44 in this pathway, lead to many questions that will need investigating.

The enrichment of MYC-target gene products is of particular interest, as MYCN and C-MYC play important roles in the pathophysiology of neuroblastoma. We recently found that USP44 promotes a MYC-like gene expression program in neuroblastoma cells and in murine fibroblasts [2]. In that study, the mechanism involved regulation of gene transcription by deubiquitination of histone H2BK120. The observation that MYC-target gene products also enriched in the USP44 proximity proteome may suggest that USP44 may promote MYC-like signaling through a post-translational mechanism as well. This notion is supported by recent work that showed a direct interaction between USP44 and C-MYC resulting in the deubiquitination and stabilization of C-MYC [39,40]. This observation requires further study.

## Data Availability

Proteomics data generated are provided in Supplemental Table S1. All clinically annotated human datasets used in the study are publically available through the R2: Genomics and visualization platform as described in the Section 2.

## Supplementary Materials

The following supporting information can be downloaded at: https://www.mdpi.com/article/10.3390/biomedicines12122901/s1, Figure S1. STRING proximity proteome network for USP44. Figure S2. High levels of BRCA2 predict poor outcomes in neuroblastoma. Table S1. Enriched proteins.; Table S2. Enriched Cancer Genes.
