# Supplementary material for: Proximity Proteomics Reveals USP44 Forms a Complex with BRCA2 in Neuroblastoma Cells and Is Required to Prevent Chromosome Breakage
Source: Biomedicines. 2024 Dec 20;12(12):2901. doi: 10.3390/biomedicines12122901 (PMC11727000; doi:10.3390/biomedicines12122901)
Supplement: Supplementary file 1 [file biomedicines-12-02901-s001.zip › biomedicines-3320857-supplementary.pptx]

## Slide 1
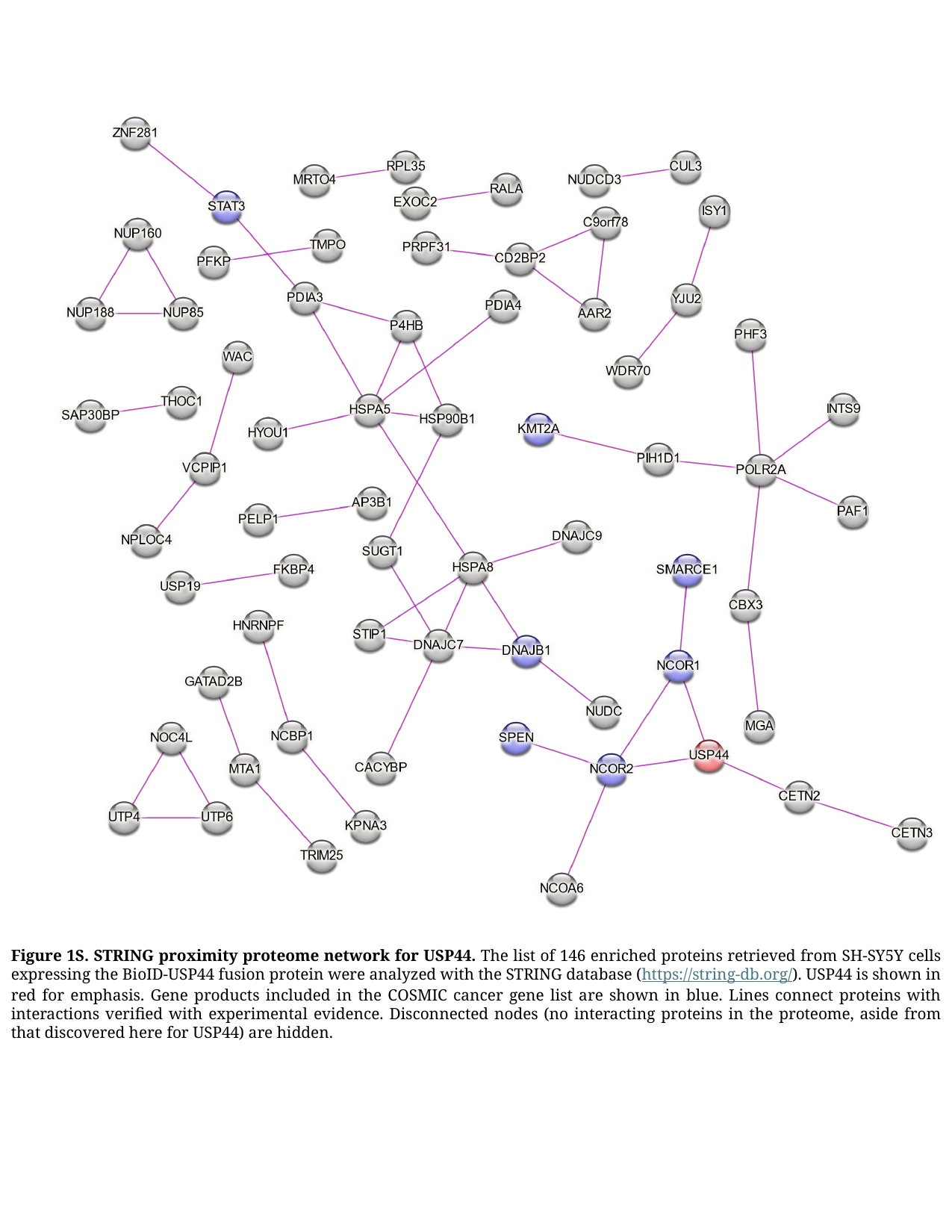

Figure 1S. STRING proximity proteome network for USP44. The list of 146 enriched proteins retrieved from SH-SY5Y cells expressing the BioID-USP44 fusion protein were analyzed with the STRING database (https://string-db.org/). USP44 is shown in red for emphasis. Gene products included in the COSMIC cancer gene list are shown in blue. Lines connect proteins with interactions verified with experimental evidence. Disconnected nodes (no interacting proteins in the proteome, aside from that discovered here for USP44) are hidden.

## Slide 2
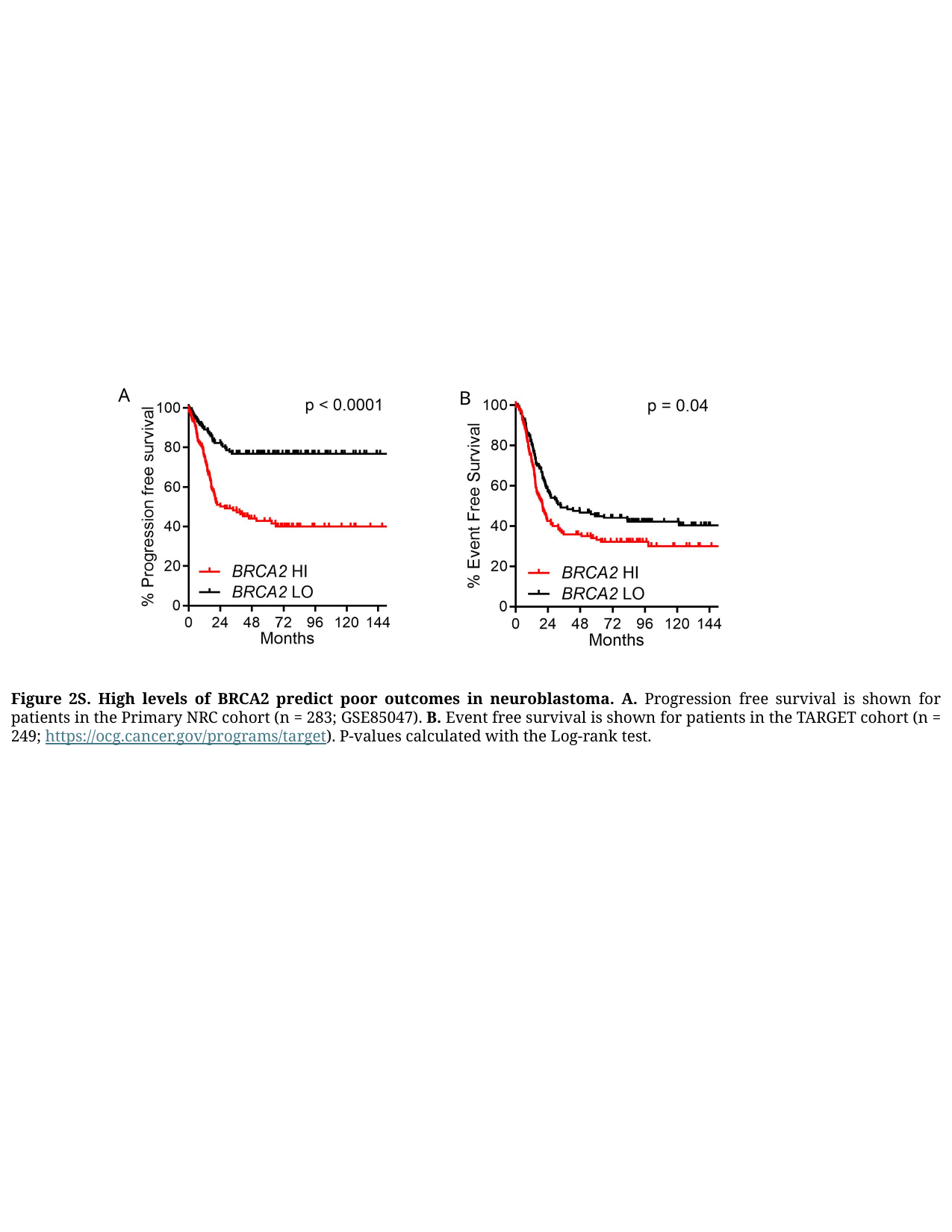

A
B
Figure 2S. High levels of BRCA2 predict poor outcomes in neuroblastoma. A. Progression free survival is shown for patients in the Primary NRC cohort (n = 283; GSE85047). B. Event free survival is shown for patients in the TARGET cohort (n = 249; https://ocg.cancer.gov/programs/target). P-values calculated with the Log-rank test.
